# Decision time and confidence predict choosers' identification performance in photographic showups

**DOI:** 10.1371/journal.pone.0190416

**Published:** 2018-01-18

**Authors:** Melanie Sauerland, Anna Sagana, Siegfried L. Sporer, John T. Wixted

**Affiliations:** 1 Faculty of Psychology and Neuroscience, Department of Clinical Psychological Science, Maastricht University, Maastricht, The Netherlands; 2 Department of Psychology and Sports Science, University of Giessen, Giessen, Germany; 3 Department of Psychology, University of California, San Diego, United States of America; University College London, UNITED KINGDOM

## Abstract

In vast contrast to the multitude of lineup studies that report on the link between decision time, confidence, and identification accuracy, only a few studies looked at these associations for showups, with results varying widely across studies. We therefore set out to test the individual and combined value of decision time and post-decision confidence for diagnosing the accuracy of positive showup decisions using confidence-accuracy characteristic curves and Bayesian analyses. Three-hundred-eighty-four participants viewed a stimulus event and were subsequently presented with two showups which could be target-present or target-absent. As expected, we found a negative decision time-accuracy and a positive post-decision confidence-accuracy correlation for showup selections. Confidence-accuracy characteristic curves demonstrated the expected additive effect of combining both postdictors. Likewise, Bayesian analyses, taking into account all possible target-presence base rate values showed that fast and confident identification decisions were more diagnostic than slow or less confident decisions, with the combination of both being most diagnostic for postdicting accurate and inaccurate decisions. The postdictive value of decision time and post-decision confidence was higher when the prior probability that the suspect is the perpetrator was high compared to when the prior probability that the suspect is the perpetrator was low. The frequent use of showups in practice emphasizes the importance of these findings for court proceedings. Overall, these findings support the idea that courts should have most trust in showup identifications that were made fast and confidently, and least in showup identifications that were made slowly and with low confidence.

## Introduction

The identity of a perpetrator is commonly established by means of an identification procedure, for example a police lineup or showup. While witnesses can play a crucial role in police investigation, it is well-established that eyewitnesses can err, as evidenced by wrongful convictions in which eyewitness testimony played a key role ([1], innocenceproject.org). Although proper lineup construction and administration can decrease error rates [2], the risk of false identifications continues to be a major concern in the field. This concern highlights the need for measures that can help to evaluate the accuracy of identification decisions.

The most promising assessment variables (or postdictors) of eyewitness identifications from a lineup are decision time and post-decision confidence. In a lineup, a suspect is presented to the witness together with a number of foils (individuals who are known to be innocent). Possible lineup outcomes include the selection of the suspect (who may or may not be the perpetrator), the selection of a foil, or a rejection. Theory suggests that confidence and decision times index the degree of match between a presented stimulus and the individual’s memory of a previously viewed stimulus [3–6]. According to the standard signal detection model of recognition memory, strong memories and high levels of match should be associated with accurate, confident, and fast decisions, whereas weaker memories and lower levels of match should be associated with higher error rates, lower confidence, and slower decisions [7,8]. These theories predict meaningful relationships between confidence, decision time and accuracy because all three measures gauge memory quality and the degree of match between a presented stimulus and an image in memory [9–11]. The empirical database is in support of these theoretical considerations: accurate lineup selections are made faster and with more confidence than inaccurate ones (e.g., [8,12,13,14]). The confidence-accuracy relationship is stronger in situations where a confidence judgment is collected immediately following the decisions compared to later on, for example in the court room (e.g., [2,14]). A combination of both postdictors has been shown to further improve their postdictive value [15–18]. The associations between identification accuracy and postdictors are weak for lineup rejections [19–22].

An alternative identification procedure to the lineup is the showup, which is in effect a one-person lineup. Showups are potentially problematic because they communicate the hypothesis of the administrator to the witness ([23]; cf. the lineups-as-experiments analogy,[24]). As such, showups can be construed as being in violation of rule 3 of Wells et al.’s [23] recommendations for lineups and photospreads:

“The suspect should not stand out in the lineup or photospread as being different from the distractors based on the eyewitness's previous description of the culprit or based on other factors that would draw extra attention to the suspect.” (p. 630)

Nevertheless, showups are frequently applied in the field, based on the argument that they can be conducted very quickly, even within hours following a crime, are less costly than lineups [25,26], and require less preparation (i.e., finding matching foils and testing for lineup fairness is not necessary). In the field, showups are probably applied more often than lineups [26–30]. Despite its evident suggestive nature, comparisons between lineups and showups indicate that showups don’t necessarily lead to higher false alarm rates, but in fact result in lower choosing rates than lineups [30]. More recent meta-analyses revealed a no-cost pattern [31].

While many are not in favor of showups, given its lack of protection to the suspect, its frequent use in the field establishes a need for research that enables investigators and legal practitioners to assess the accuracy of showup decisions. Key et al. [32] addressed this issue by combining decision time and confidence as postdictors of showup selections. Their results suggest that conducting a showup may be preferable to a biased lineup, as long as selections are made both quickly and with confidence.

Given the regularity of showup use in the field, it is remarkable that Key et al. [32] is the only study that looked at the postdictive value of decision time and confidence for showup selections in combination. In addition, a handful of studies investigated the association between post-decision confidence and accuracy of showup selections. From a theoretical perspective, there is little reason to expect differences between showup and lineups in the confidence-accuracy and decision time-accuracy relationships for positive identifications (although differences can be expected and have been reported for lineup rejections; see [33]). It is therefore surprising, at first sight that the effect sizes in these studies exhibit remarkable heterogeneity, including null [29], small ([26] Experiment 3,[34] Experiment 3,[35] Experiment 1); moderate ([26] Experiment 1,[34] Experiment 2,[36]), and large effects ([35] Experiment 2,[37]). Closer inspection of these studies suggests that moderator effects may be accountable for these results. For example, a large proportion of Dysart et al.’s participants were intoxicated, a condition that is known to deflate the confidence-accuracy relationship [38]. Yarmey et al. ([35], Experiment 1) report confidence-accuracy relationship across different delays (no delay vs. 30 min vs. 2 hrs vs. 24 hrs delay) and clothing bias (bias vs. no bias) conditions. Under more difficult conditions (such as longer delay or shorter exposure), smaller effect sizes are in fact in line with the literature (see [39], for a discussion of factors that may affect accuracy but not confidence, [40]).

Given the dearth of literature regarding reaction times and the heterogeneity in the size of the confidence-accuracy associations across studies, we consider it essential to accumulate more data on the issue (cf. [41]), thus extending the database to different stimuli and procedures, and refining the analysis by factoring in the prior probability that a suspect is guilty. Adding to the literature in this way will eventually enable researchers to conduct meta-analyses to establish the true effect size and moderators of the confidence-accuracy relationship [42,43]. In the current study we contribute to the literature on postdicting showup decisions using confidence-accuracy characteristic curves and Bayesian analyses, which allowed us to investigate the postdictive value of confidence and decision time as a factor of different base rates, or prior probabilities, that the suspect is the perpetrator [8,44]. In most lab studies, a base rate of 50% (i.e., 50% target-present and 50% target-absent lineups or showups) is employed. Although it is unknown what this figure would look like in the real world, it is likely that it varies across different legislations and police departments. Base rate probably also vary with the precision of the description of the perpetrator given to the police. For example, the prior probability that a given suspect is guilty would likely be low for vague descriptions like "he was a male between 20 and 40 years of age" because many people in the vicinity of the crime would match that description. By contrast, the prior probability of guilt would likely be higher for more specific descriptions like "he was a bald male, about 30 years old, with a handlebar mustache and a neck tattoo" because very few people in the vicinity of the crime would match that description. Importantly, the postdictive value of confidence following a lineup decision varies as a factor of this base rate [8], with higher target-presence base rates being associated with higher postdictive value compared to lower target-presence base rates. To this end, we analyzed the chooser data collected (but not reported) by Sauerland et al. [33]. We expect to find a similar pattern of results for showups. To our knowledge, this will be the first study to provide such data for showups.

## Method

Upon publication, all data will be publically available using the following link: http://hdl.handle.net/10411/V37C99.

### Ethics statement

This study was approved by the ethics committee of the Faculty of Psychology and Neuroscience of Maastricht University (ECP-85 01-10-2009-v2) and follows the rules stated in the Declaration of Helsinki. Participants provided oral and/or written consent. Oral consent was sufficient because data were analyzed anonymously.

### Participants

Participants (*N* = 384; 167 men, 217 women; 18–60 years, *Mdn* = 26.5 years) were students (71%), worked in professions excluding academics (24%), or were academics (5%).

### Design

Participants were randomly assigned to one of two experimental conditions (target-presence thief and victim showup were either presented as thief present/ victim absent or thief absent/ victim present, respectively).

## Materials

### Photo showups and innocent suspect assignment

Four students (2 men, 2 women, age 20–33 years) served as targets. Showups consisted of one photograph, juxtaposed by a “not present” and a “don’t know” option at the side. The size of the photos on the screen was 15.7cm x 14.6cm.

Innocent suspects in target-absent showups were chosen to match the description of the target. For each target, we took pictures of 12 persons who matched their general description. Those, together with the picture of the target were presented to 55 mock witnesses. The photos that were chosen most often apart from the target were selected to be the innocent suspects. Furthermore, the physical similarity between the innocent suspects and the targets were rated on a 7-point Likert scale by 15 different persons. On average, target-innocent suspect similarity was moderate (thieves: *M*s = 4.17 and 2.75 [*SD*s = 1.59, 1.42], victims: *M*s = 3.67 and 3.25 [*SD*s = 1.61, 1.36]).

### Stimulus films

The two stimulus films involved four different actors each (thief, victim, two bystanders) and depicted the theft of a wallet in a student cafeteria (duration: 5:05 and 3:15 min, respectively). In film 1, the thief was male, the victim female; in film 2, it was the other way round (but with different actors). Each person could be seen for at least 89 s, and there were close-ups of all targets varying between 2 and 9 s. The close-up filled, on average, 11.7% (*SD* = 5.8) of the screen. All targets could be seen from frontal and side views. The action can be described as follows: three students sit together talking about the summer break. After a while, a fourth student (the thief), unfamiliar to the former three, sits down next to them and reads a book. When the future victim gets up to get some coffee, the thief steals the wallet of the victim without the other two students noticing. After leaving the table, a close-up view of the perpetrator going through the wallet follows. When the victim returns s/he realizes that the wallet was stolen.

### Procedure

Participants were tested individually or in groups of two. All instructions and the showups were presented on a computer screen using SuperLab 1.75 (*www.cedrus.com*). Participants were informed that the experiment dealt with witness statements, not mentioning the topic of person identification. After viewing one of the two films participants completed a 30 min filler task consisting of 40 general knowledge questions. Participants then viewed showups of the thief and the victim, with the thief showups always presented first. Participants could make a selection, state that the person seen in the film was not present in the showup, or indicate that they did not know. Participants were warned that the person from the film may or may not be present before each showup. Decision times were measured through the software. Before and after each showup, participants indicated their pre- and post-decision confidence on an 11-point scale ranging from 0% to 100%. No post-decision confidence ratings were obtained following don't know responses. Finally, participants were thanked and debriefed.

## Results

This paper focuses on positive identification decisions (i.e., choosers), with one exception: for the Bayesian analyses, hit and false alarm rates were computed, taking into account non-selections as well. The sample of nonchoosers and the rejection data referred to were described in Sauerland et al. [33]. Following other researchers [17,20,45], we report results for the thief and victim showup combined. This is in accordance with the idea of stimulus sampling [17,46] and ensures a more stable representation of the associations displayed. For decision time, inferential analyses were conducted on log-transformed data (i.e., log base 10) due to significant positive skewness and kurtosis in the decision time distribution. Means are reported for back-transformed values.

### Overview

Selections were made 274 times, of which 241 were correct (i.e., occurred in target-present showups). Thus, the correct positive identification rate was 88% (241 / 274 = .88), and significantly higher than chance level (i.e., 50%), *t*(273) = 19.27, *p* < .001. The resulting average selection rate for target-present showups was 241/384 (i.e., 62.8% hits) and 33/384 (i.e., 8.6% false identifications) for target-absent showups, leading to an average selection rate of 35.7%. Descriptive statistics of decision time and confidence can be found in Table 1. As expected, a significant negative decision time-accuracy relationship, *r*(272) = -.19, *p* = .002, and a positive post-decision confidence-accuracy relationship was found, *r*(272) = .19, *p* = .002, displaying small to moderate effect sizes.

**Table 1:** Means, SDs and 95% CIs of choosers’ decision accuracy, post-decision confidence, and decision time.

	*M*	*SD*	*95% CI*
Decision accuracy	88.0%	32.6	84.1–91.8%
Post-decision confidence	80.9%	21.1	78.4–83.4%
Decision time	7.5 s	7.6 s	6.6–8.4 s

CI = Confidence interval.

### Combining postdictors

**Fig 1:**
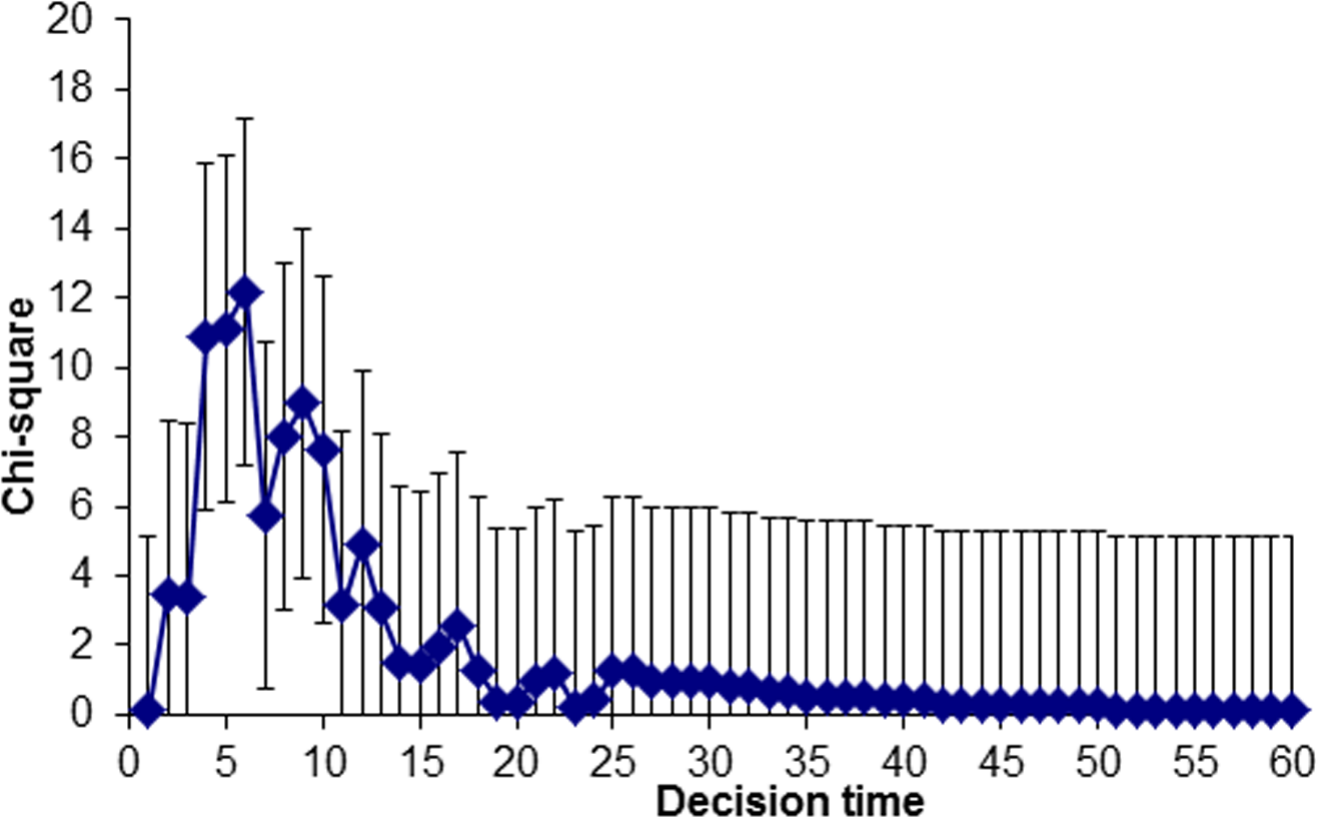
Plot of chi2-values (and 95% confidence interval) by decision time boundary for choosers.

To combine the two postdictors, we established the optimum decision time boundary, which is the time boundary that optimally discriminated between correct and incorrect choosers [47]. It was computed based on the 2 (accuracy: correct vs. incorrect) x 2 (time boundary: faster or equal vs. slower) contingency tables with the time boundary set at each integer value (i.e., 1 s, 2 s, etc.). The plot of *chi2*-values by time boundary is presented in Fig 1. The optimum time boundary was at 6 s, *chi2*(1, *N* = 274) = 12.19, *p* < .001, *phi* = -.21. This is similar to the 8-second boundary observed by Key et al. [32] for showup choosers and the 7-second boundary reported by Sauerland et al. [33] for showup nonchoosers. The mean proportion of correct selections made before the optimum time boundary was 93.8% and 79.8% afterwards (with an average accuracy of 88.0%). For post-decision confidence, we determined confidence ratings below 90% as “low” and confidence ratings between 90 and 100% as high (cf. [18]). Confident decisions were accurate in 92.4% of the cases, non-confident decisions in 83.1%.

**Fig 2:**
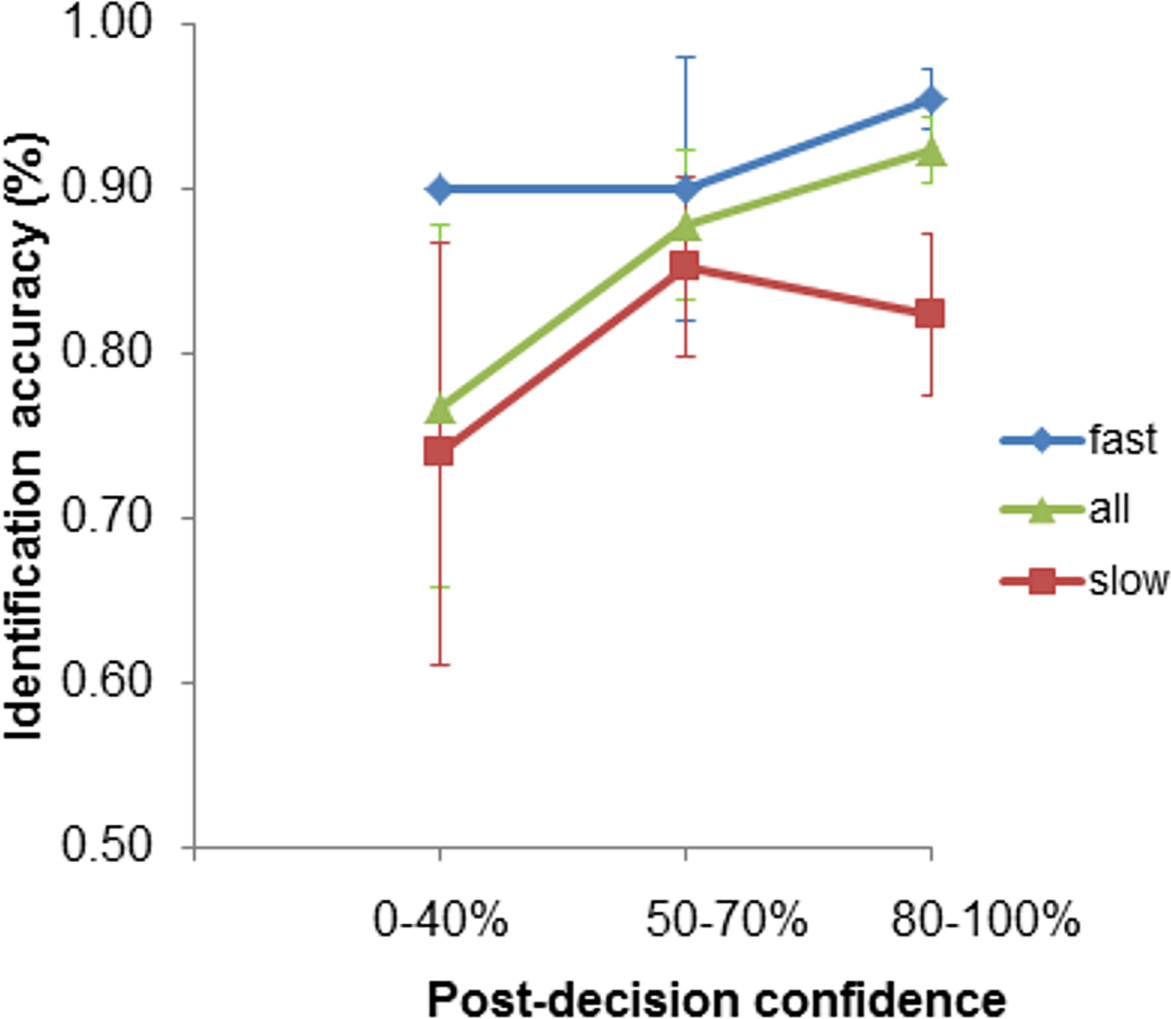
Confidence-accuracy characteristic curves (and 95% confidence intervals) for all, fast, and slow chooser decisions.

One way to present the data is by examining variations in calibration with identification decision time by dividing participants into fast and slow groups, based on the decision time boundary analyses. However, the sample sizes for fast and slow choosers were too small to produce stable estimates for each calibration curve and the associated statistics (cf. [15]). We therefore plotted confidence-accuracy characteristic (CAC) curves (see Fig 2) for fast and slow choosers (cf. [48]). The curve for fast decisions consistently lies above the curves for slow decisions and accuracy for more confident decisions was higher than for less confident decisions. The curves also suggest some additive value of confidence and decision time for postdicting identification accuracy, especially at the highest level of confidence. That is, fast identifications were more likely to be accurate at every level of confidence (although the difference was small for medium level of confidence). Likewise, more confident showup identifications were more likely to be accurate for fast than slow decision times.

### Considering base rates of target-presence and postdictors

**Fig 3:**
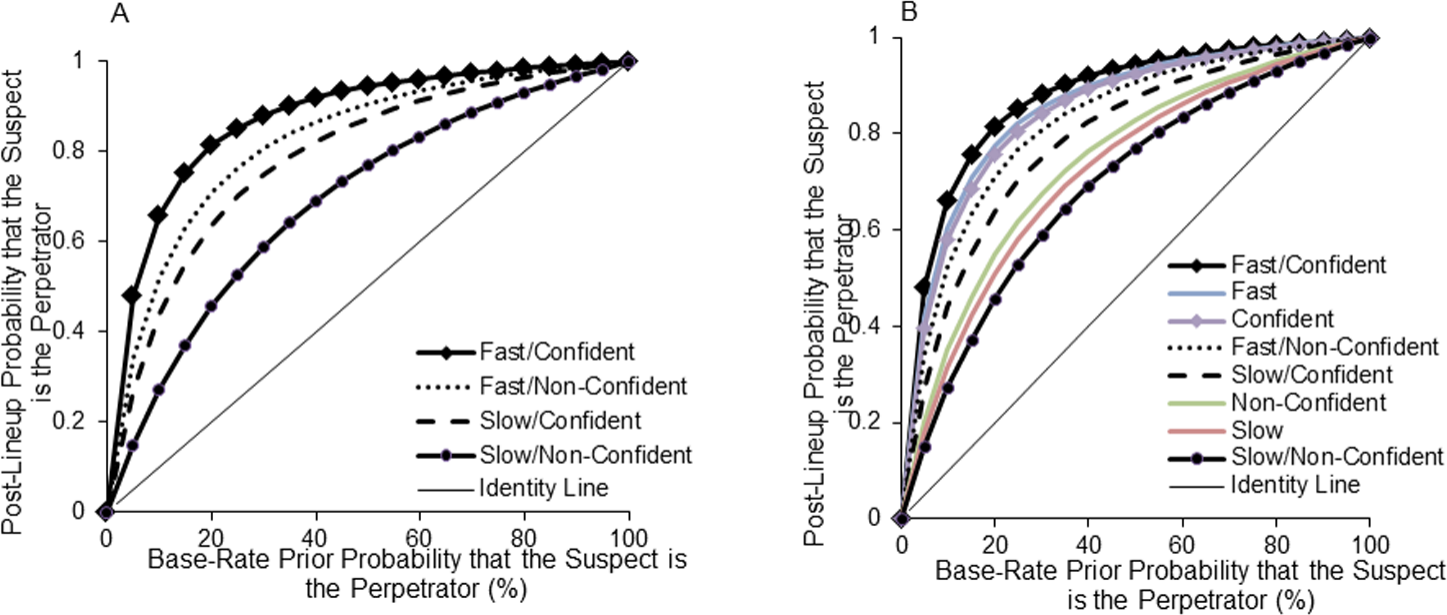
Post-lineup probability that the suspect is the perpetrator as a function of the base rate of target-presence for postdictor combinations (panel A) and postdictor combinations and individual postdictors (panel B).

Next, we addressed the possible concern that the relationships between postdictors and accuracy may hold only for certain base rates or prior probabilities that the suspect is the perpetrator. Fig 3*A* maps the probability that a suspect identification was accurate (i.e., that the suspect is the perpetrator) across all possible base rate values from 0% (all showups displayed an innocent suspect) to 100% (all showups displayed a guilty suspect; see 8, for a short tutorial on this Bayesian approach). One curve was created for each postdictor combination, that is, for fast-non-confident, slow-non-confident, fast-confident, and slow-non-confident identifications. In Fig 3*B*, the curves for fast, slow, confident, and non-confident decision were added. The identity line shows where the data would fall if an identification was non-diagnostic.

Three important observations can be made from Fig 3A and 3B. First, all curves are above the identity line, indicating that identifications were diagnostic of guilt. More importantly, as expected, the height of the curves for confident or fast decisions is far above that of the curves for less confident or slow decisions. Third, the combination of both postdictors improves postdiction even more: the curve for fast-confident decisions is the highest of all, whereas the curve for slow-non-confident decisions is the lowest of all.

Note that the probability that the identified suspect is the perpetrator for decisions that are made fast and with high confidence remains high (above 90%) until the base rate drops below 35%. For slow-less confident decisions, accuracy drops below 90% at a base rate of 75%. Or in other words, whereas fast-confident decisions were still highly accurate (90%) at a base rate of a mere 35%, slow-non-confident decisions were much less accurate (65%) at this base rate.

## Discussion

It was the aim of this paper to contribute to the literature on postdicting showup decisions by means of confidence and decision time. Although there is little reason to believe that the association between postdictors and identification accuracy should be fundamentally different for lineups vs. showups, it is remarkable that so few studies reported on this relationship for showups. More specifically, only one study has looked at the link between decision time and showup identification accuracy to date and a hand full of studies investigated the confidence-accuracy relationship for showups. This stands in vast contrast to the dozens of studies that report on these relationships for lineups (e.g., [8,21]). We therefore set out to test the individual and combined value of decision time and post-decision confidence for diagnosing the accuracy of positive showup decisions. We expected to find analogous results to the lineup literature, namely a negative decision time-accuracy relationship and a positive confidence-accuracy relationship for choosers. Furthermore, we predicted that combinations of both postdictors would lead to more accurate decision classifications than each postdictor alone.

The results supported our hypotheses. Specifically, we found a negative decision time-accuracy and a positive post-decision confidence-accuracy correlation for showup selections. The confidence-accuracy characteristic curves demonstrated the expected additive effect of combining both postdictors: fast identifications were more likely to be accurate at every level of confidence (although the difference was small for medium level of confidence) and more confident showup identifications were more likely to be accurate for fast than slow decision times. The Bayesian analyses, taking into account all possible base rate values from 0% to 100%, led to similar observations: *Fig 3A and 3B* shows that fast and confident identification decisions were more diagnostic than slow or less confident decisions, with the combination of both being most diagnostic for postdicting accurate (fast and confident) or inaccurate (slow and non-confident) decisions.

Another important observation concerns the finding that the base rate at which a suspect is guilty affects the postdictive value of assessment variables. This is in line with previous analyses concerning lineups [8]. When a lineup is used, the base rate of guilt can be increased by ensuring that there is independent evidence against a suspect (beyond matching the description of the perpetrator) before placing that suspect in a lineup [49]. By contrast, when a showup is used in the immediate aftermath of a crime, there is little time for an investigation to yield independent evidence, so the suspect's inclusion in the identification test will likely be based largely on the physical description of the perpetrator provided by the eyewitness. Under such conditions, it seems reasonable to suppose that the more precise the description, the higher the prior probability of guilt. The results shown in Fig 3 illustrate how important this consideration is–over and above the confidence and timing of the identification–in determining the posterior probability that the identified suspect is guilty.

As with all lab-based research, it is difficult to assess the degree to which the conditions of our experiment correspond to the typical showup in the real world. For example, on those occasions when the police detain an innocent suspect for a showup, how similar in appearance is that suspect, on average, to the true perpetrator? The more similar they are, the more it will reduce the accuracy of even fast identifications made with high confidence. In the extreme, for example, if the police detain the identical twin of the perpetrator, a witness with a clear memory of the perpetrator may make a fast, high-confidence (yet mistaken) identification of that innocent suspect. Considerations such as these may help to explain differences in high confidence accuracy for showups in various studies. For example, Eisen et al. [50] recently reported a field-simulation study of showup accuracy. Their Experiment 1 varied the similarity of the innocent suspect and the perpetrator (high vs. low), but it included only a target-absent condition, so accuracy could not be computed. Their Experiments 2 and 3 included both target-present and target-absent showups, but used only the high-similarity innocent suspect. Under those conditions, they found that high-confidence accuracy fell between 75% and 80% correct (though they did not separately report the accuracy of fast high-confidence identifications, which presumably would have been more accurate). Although studies differ in high-confidence accuracy for showups, they agree that confidence is strongly indicative of accuracy.

As a final note we would like to emphasize that, in general, the diagnostic value of fair multiple person lineups should be valued more highly than showups. Thus, although it is sometimes essential for the police to conduct showups in the early stages of an investigation (and our work suggests that the obtained information can be reliable), investigators should not conduct showups if a fair lineup is feasible. When a showup is essential, the probable development of specific apps may facilitate the preservation of confidence and decision times for showups and street identifications for later assessment in the future.
